# Association of chemerin mRNA expression in human epicardial adipose tissue with coronary atherosclerosis

**DOI:** 10.1186/1475-2840-10-87

**Published:** 2011-10-07

**Authors:** Xiuying Gao, Shuhua Mi, Fuzhuang Zhang, Fengying Gong, Yongqiang Lai, Feng Gao, Xiaoxia Zhang, Linjie Wang, Hong Tao

**Affiliations:** 1 Department of Endocrinology, The Key Laboratory of Remodeling-related Cardiovascular Diseases, Beijing Anzhen Hospital, Capital Medical University, Ministry of Education, Beijing, 100029, China; 2 Department of Special Care, Beijing Anzhen Hospital, Capital Medical University, Beijing, 100029, China; 3 Department of Endocrinology, Peking Union Medical College Hospital, Peking Union Medical College, Chinese Academy of Medical Sciences, Beijing, 100730, China; 4 Department of Cardiac Surgery, Beijing Anzhen Hospital, Capital Medical University, Beijing, 100029, China

**Keywords:** Epicardial adipose tissue, Chemerin, Adipokine, Atherosclerosis

## Abstract

**Background:**

Growing evidence suggests that epicardial adipose tissue (EAT) may play a key role in the pathogenesis and development of coronary artery disease (CAD) by producing several inflammatory adipokines. Chemerin, a novel adipokine, has been reported to be involved in regulating immune responses and glucolipid metabolism. Given these properties, chemerin may provide an interesting link between obesity, inflammation and atherosclerosis. In this study, we sought to determine the relationship of chemerin expression in EAT and the severity of coronary atherosclerosis in Han Chinese patients.

**Methods:**

Serums and adipose tissue biopsies (epicardial and thoracic subcutaneous) were obtained from CAD (n = 37) and NCAD (n = 16) patients undergoing elective cardiac surgery. Gensini score was used to assess the severity of CAD. Serum levels of chemerin, adiponectin and insulin were measured by ELISA. Chemerin protein expression in adipose tissue was detected by immunohistochemistry. The mRNA levels of chemerin, chemR23, adiponectin and TNF-alpha in adipose tissue were detected by RT-PCR.

**Results:**

We found that EAT of CAD group showed significantly higher levels of chemerin and TNF-alpha mRNA, and significantly lower level of adiponectin mRNA than that of NCAD patients. In CAD group, significantly higher levels of chemerin mRNA and protein were observed in EAT than in paired subcutaneous adipose tissue (SAT), whereas such significant difference was not found in NCAD group. Chemerin mRNA expression in EAT was positively correlated with Gensini score (r = 0.365, *P *< 0.05), moreover, this correlation remained statistically significant (r = 0.357, *P *< 0.05) after adjusting for age, gender, BMI and waist circumference. Chemerin mRNA expression in EAT was also positively correlated with BMI (r = 0.305, *P *< 0.05), waist circumference (r = 0.384, *P *< 0.01), fasting blood glucose (r = 0.334, *P *< 0.05) and negatively correlated with adiponectin mRNA expression in EAT (r = -0.322, *P *< 0.05). However, there were no significant differences in the serum levels of chemerin or adiponectin between the two groups. Likewise, neither serum chemerin nor serum adiponectin was associated with Gensini score (*P *> 0.05).

**Conclusions:**

The expressions of chemerin mRNA and protein are significantly higher in EAT from patients with CAD in Han Chinese patients. Furthermore, the severity of coronary atherosclerosis is positive correlated with the level of chemerin mRNA in EAT rather than its circulating level.

## Background

Currently, obesity and accumulation of visceral adipose tissue have been considered as the important risk factors for metabolic syndrome and coronary artery disease (CAD) [1]. Adipose tissue is now recognized as an active endocrine organ expressing and secreting several inflammatory mediators and cytokines, also known as adipokines, which participate in the development of atherosclerosis [2]. Epicardial adipose tissue (EAT) is a type of visceral fat [3]. Most recently, particular research interests have focused on EAT due to its close proximity to coronary arteries and the plausibility that the adipokines derived from it may have direct effects on each layer of the coronary wall via paracrine and vasocrine pathways [4]. Previous studies have revealed that atherosclerotic intimal lesions are not seen in the coronary arterial segments absent of EAT or covered by myocardium [5], suggesting EAT may play a role in the development of coronary atherosclerosis. Subsequent studies have demonstrated that significantly lower level of adiponectin and significantly higher levels of inflammatory cytokines exist in the EAT of patients with CAD [6,7]. Recently, some evidence also proves that CAD is associated with increased chemokines production by EAT [8-11]. In addition, infiltration of macrophages are increased [12] and inflammatory pathways mediated by nuclear factor-κB and Janus kinase (JNK) are activated in EAT in the presence of CAD [13]. All these data above suggest EAT, due to its regional pro-inflammatory properties, may play an important role in the pathogenesis of CAD.

Chemerin, also known as tazarotene-induced gene 2 (TIG2) or retinoic acid receptor responder 2 (RARRES2), functions as a chemotactic protein that binds to the G protein-coupled receptor, CMKLR1 (chemR23), in humans [14]. Recently, it has been identified as a novel adipokine owing to its high expression level in white adipocytes. Chemerin has been reported to be involved in modulating immune responses and glucolipid metabolism. Furthermore, some studies have shown the strong association of serum chemerin level with markers of inflammation and components of the metabolic syndrome [15]. Therefore, the double role of chemerin in inflammation and metabolism may provide an interesting link between obesity, inflammation and atherosclerosis. However, the association of chemerin with atherosclerosis has not yet been proved. In this study, we sought to determine the relationship of chemerin expression in human EAT and the severity of coronary atherosclerosis.

## Methods

### Subjects

Between October 2009 and March 2010, a total of 53 Han Chinese patients who underwent elective cardiac surgery were enrolled in the study. Exclusion criteria included non ischemic cardiomyopathy, liver or renal failure, neoplastic disease, thyroid or adrenal gland disease, acute infectious disease, the aged (> 80 years) or patients taking corticosteroids, oral contraceptives, or psychotropic drugs. Patients were divided into CAD group and NCAD group according to the results of selective coronary angiography. CAD was defined as the presence of stenoses of greater than 50% of the luminal diameter for at least one of the three major coronary arteries. All CAD patients underwent elective coronary artery bypass graft (CABG) surgery. All NCAD patients received elective open-heart surgery for valvular replacement or atrial septal defect closure. These patients had no clinical signs of CAD and did not show significant coronary stenoses (≥50%) in the pre-operative coronary angiographic examination.

This study was approved by the ethics committee of Beijing Anzhen Hospital of Capital Medical University. This study complied with the declaration of Helsinki with the written informed consent obtained from all subjects prior to enrolment.

### Clinical data collection

Clinical data were obtained upon admission to hospital before surgery. Demographic data, medical history, and medications being used before surgery were recorded. In addition, height, body weight, waist circumference and blood pressure were measured. Then body mass index (BMI) was calculated as weight (kg) divided by square of height (m^2^).

### Gensini score assessment

Angiographic analyses were carried out by two experienced interventional cardiologists who were blinded to the study protocol according to the result of coronary angiography within six months prior to surgery. Then Gensini score was calculated to assess the severity of coronary atherosclerosis according to the method described in the literature [16].

### Blood samples detection

Peripheral venous blood samples were collected after overnight fasting on the day after admission, and serum glucose, lipid profiles, high sensitivity C-reactive protein (hsCRP), insulin and hemoglobin A_1_C (HbA_1_C) were analyzed in central laboratory of Beijing Anzhen Hospital. Insulin sensitivity was estimated by homeostasis model assessment of insulin resistance (HOMA-IR) calculated as fasting glucose (mmol/L) × fasting insulin (μU/mL)/22.5.

Central venous blood was drawn before cardiopulmonary bypass during the operation. Then serum was stored in aliquots at -80°C until being analyzed. Serum levels of chemerin and adiponectin were determined by commercially available enzyme-linked immunosorbent assay (ELISA) kits (Adipobiotech, Beijing, China).

### Adipose tissue acquisition

Adipose biopsy samples were obtained prior to the initiation of cardiopulmonary bypass from areas that had not previously been injured mechanically or cauterized. EAT biopsies (average 0.5 g) were taken near the proximal right coronary artery, and subcutaneous adipose tissue (SAT) samples were collected from the site of the chest incision. The specimens were rinsed with normal saline and divided into two portions. After removal of visible blood vessels, one portion was frozen immediately in liquid nitrogen and stored at -80°C for RNA isolation, another was immersed in neutralized formalin for immunohistochemical analysis.

### Immunohistochemistry

Paraffin-embedded tissue sections were deparaffined and rehydrated in descending grades of alcohol and stained with hematoxylin and eosin. Selected serial sections were subjected to immunohistochemistry with the Histostain-Plus Kit LAB-SA Detection System (Invitrogen, USA) according to the manufacturer's protocol. Briefly, sections were incubated in 3% H_2_O_2 _for 20 minutes followed by blocking with normal goat serum. After they were washed in PBS, sections were incubated with primary antibodies (Chemerin, 1:200, Adipobiotech, Beijing, China) overnight at 4°C in a moisture chamber. Afterward, the slides were incubated with biotinylated secondary antibodies for 10~15 minutes followed by avidin-biotin for 15 minutes. Sections were then exposed to DAB and counterstained with hematoxylin. Negative controls were carried out omitting the primary antibody. Brightfield images were observed with a light microscope and digital images were recorded. Positive staining for chemerin was brown. Expression of chemerin was quantified by calculating the integrated optical density (IOD) of positive staining tissue via Image-pro plus software. The IOD of each tissue section was calculated from eight different 400 magnified fields.

### RNA extraction and Reverse Transcription-Polymerase Chain Reaction (RT-PCR)

Total RNA was extracted from adipose tissue samples using Trizol reagent (Invitrogen, USA). The concentration and purity of isolated RNA were assessed by measuring the optical density at 260 nm (OD260) and 280 nm (OD280), and the integrity of RNA was also determined by visualisation of 18S and 28S ribosomal bands. 1 μg of RNA from each sample was reverse transcribed using oligo(dT)_15 _and M-MLV Reverse Transcriptase (Promega, USA), according to the manufacturer's instructions. An aliquot of 2 μL of the resulting cDNA was used for semiquantitative PCR. PCR was carried out in a volume of 25 μL containing 12.5 μL 2 × PCR-Mix, 1 μL 10 μmol/L each primer, and sterile water. The primers were designed using the Primer 5.0 software program, with their sites spanning introns, so quantitation of the reaction was not affected by the presence of genomic DNA. The primers used for amplification were shown in Table 1. Apart from the optimal annealing temperature and number of cycles of amplification, other conditions were the same in all PCR reactions. PCR was performed under the following conditions: 3 minutes for an initial denaturation at 94°C, followed by different cycles (30 s for denaturation at 94°C, 30 s for annealing and 50 s for extension at 72°C), and 5 minutes for a final extension at 72°C. The number of PCR cycles in each system was chosen within linear phase in our preliminary trial to ensure the accuracy of semiquantitative analysis. A 5 μl aliquot of PCR products was then analyzed using 1.5% agarose gel electrophoresis. Band intensity for the target gene was quantified by densitometry using Scion Image software and normalized to β-actin mRNA level to determine relative mRNA expression of the target gene.

**Table 1:** Primers for RT-PCR reaction

Gene title	Accession number	Primer sequence	Cycle	Ta (°C)	Product (bp)
Chemerin	NM_002889.3	F: 5'-GAAGAAACCCGAGTGCAAAG-3'	28	56	229
		R: 5'-CTTGGAGAAGGCGAACTGTC-3'			
ChemR23	NM_001142343.1	F: 5'-CTCCCAATCCATATCACCTA-3'	32	58	543
		R: 5'-GCAGAGGAAGAAGGTAATGA-3'			
Adiponectin	NM_001177800.1	F: 5'-CTCCTCCTCACTTCCATTCTG-3'	35	58	310
		R: 5'-TTTCACCGATGTCTCCCTTA-3'			
TNF-α	NM_000594.2	F: 5'-TTCTGCCTGCTGCACTTTGGA-3'	40	60	592
		R: 5'-GGCGTTTGGGAAGGTTGGATG-3'			
β-actin	NM_001101.3	F: 5'-AGGTCATCACCATTGGCAAT-3'	26	58	357
		R: 5'-ACTCGTCATACTCCTGCTTG-3'			

Abbreviations: TNF-α, tumor necrosis factor-α; Ta, annealing temperature.

### Statistical analysis

Variables such as serum adiponectin, hsCRP and triglyceride with skewed distribution were log transformed.

Continuous variables with normal distribution were expressed as mean ± SD, means were compared by paired or unpaired Student's t-test, as appropriate. Whereas for data with skewed distribution, Mann-Whitney U test was used. Categorical variables were presented as percentages and were analyzed by Chi-squared test. Associations between the levels of adipokines in EAT and serum and clinical variables were determined with the Pearson or Spearman correlation coefficients. Partial correlation analysis was performed to evaluate adjusted association between chemerin mRNA level in EAT and Gensini score. All statistical analyses were performed using SPSS 16.0 software, the P-value < 0.05 was considered statistically significant.

## Results

### Patient characteristics

The baseline characteristics of the two groups were shown in Table 2. In the CAD group (n = 37), 30 were men, and mean age was 59.1 ± 8.0 years. In the NCAD group (n = 16), 12 were men, and mean age was 54.3 ± 9.4 years. As expected, the majority of patients undergoing CABG surgery had multi-vessel lesions, moreover, obesity and hypertension were more prevalent in the CAD group than in the NCAD group. There were no significant difference in age, gender, diabetes, left ventricular ejection fraction, blood pressure, triglycerides, total cholesterol, LDL cholesterol, HbA_1_C, serum creatinine, urea nitrogen, uric acid, medications treatment such as angiotensin converting enzyme inhibitors/angiotensin II type 1 receptor blockers (ACEIs/ARBs), oral hypoglyceimic agents, insulin and antibiotics between the two groups.

**Table 2:** Baseline characteristics of CAD and NCAD groups before surgery

	CAD (n = 37)	NCAD (n = 16)	P
Age (years)	59.1 ± 8.0	54.3 ± 9.4	NS
Male (%)	30 (81.1)	12 (75.0)	NS
Hypertension (%)	27 (73.0)	3 (18.8)	< 0.001
T_2 _DM (%)	11 (29.7)	1 (6.2)	NS
BMI (kg/m^2^)	27.1 ± 3.4	24.3 ± 2.6	0.004
Waist circumference (cm)	94.7 ± 9.2	85.5 ± 9.8	0.002
LVEF (%)	56.2 ± 10.1	60.1 ± 8.1	NS
Systolic blood pressure (mmHg)	129.3 ± 20.7	127.1 ± 19.5	NS
Diastolic blood pressure (mmHg)	78.1 ± 12.4	79 ± 14.0	NS
Aspirin (%)	16 (43.2)	0 (0)	0.005
Nitrates (%)	37 (100)	0 (0)	< 0.001
ACEIs/ARBs (%)	22 (59.5)	9 (56.2)	NS
Statins (%)	25 (67.6)	0 (0)	< 0.001
β-blockers (%)	35 (94.6)	3 (18.8)	< 0.001
Calcium channel blockers (%)	13 (35.1)	0 (0)	0.017
Insulin (%)	5 (13.5)	1 (6.2)	NS
Oral hypoglyceimic agents (%)	9 (24.3)	1 (6.2)	NS
Diuretics (%)	2 (5.4)	16 (100)	< 0.001
Antibiotics (%)	6 (16.2)	4 (25.0)	NS
Fasting glucose (mmol/L)	6.08 (5.25, 7.45)	5.19 (4.86, 5.51)	0.002
HbA_1_C (%)	6.3 ± 0.8	6.0 ± 0.6	NS
Fasting insulin (μU/mL)	4.67 (2.43, 45.75)	2.27 (1.77, 3.06)	0.031
HOMA-IR	1.43 (0.69, 1.32)	0.53 (0.44, 0.68)	0.013
Total cholesterol (mmol/L)	4.44 ± 1.00	4.83 ± 0.73	NS
HDL-C (mmol/L)	1.07 ± 0.24	1.25 ± 0.22	0.014
LDL-C (mmol/L)	2.68 ± 0.81	2.89 ± 0.53	NS
Triglycerides (mmol/L)	1.88 ± 1.09	1.77 ± 1.49	NS
Urea nitrogen (mmol/L)	6.82 ± 2.58	7.67 ± 2.46	NS
Serum creatinine (μmol/L)	89.9 ± 21.3	83.5 ± 20.1	NS
Uric acid (μmol/L)	380.2 ± 111.1	393.3 ± 96.8	NS
hsCRP (mg/L)	1.95 (0.98, 6.75)	1.07 (0.51, 1.66)	0.003

Data are presented as mean ± SD, median (lower quartile, upper quartile), or number (%), P value represents CAD group vs. NCAD group. Abbreviations: CAD, coronary artery disease; T_2_DM, type 2 diabetes; BMI: body mass index; LVEF, left ventricular ejection fraction; ACEIs: angiotensin converting enzyme inhibitors; ARBs: angiotensin II type 1 receptor blockers; HbA_1_C, hemoglobin A_1_C; HOMA-IR: homeostasis model assessment of insulin resistance; HDL-C, high-density lipoprotein cholesterol; LDL-C, low-density lipoprotein cholesterol; hsCRP, high sensitivity C-reactive protein; NS, non-significance.

However, patients in the CAD group presented higher levels of fasting glucose, HOMA-IR, hsCRP, and lower HDL cholesterol level compared to those in the NCAD group. As for current medications therapy, aspirin, nitrates, beta-blockers, statins, and calcium channel blockers were prescribed more often in the CAD patients than in the NCAD patients.

### Immunohistochemical analysis

**Figure 1:**
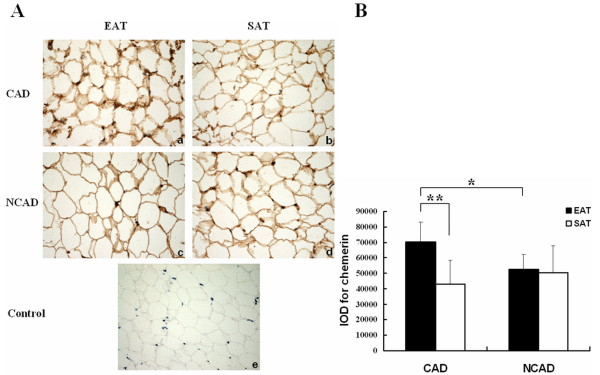
**Immunohistochemical analysis for chemerin in EAT and SAT**. (A): the representative slides of EAT and SAT are from a patient with CAD (a and b) and a patient without CAD (c and d) separately (magnified × 400). Negative control with omission of primary antibody (e). (B): the result of quantitative analysis of immunohistochemistry for chemerin in EAT and SAT of the two groups (CAD group, n = 6; NCAD group, n = 6). * indicates *P *< 0.05, EAT of CAD group vs. EAT of NCAD group. ** indicates *P *< 0.01 as determined by paired t-test, EAT of CAD group vs. SAT of CAD group. Abbreviations: EAT, epicardial adipose tissue; SAT, subcutaneous adipose tissue; IOD, integrated optical density.

We performed immunohistochemistry to illustrate chemerin expression in adipose tissue. EAT and SAT samples were collected randomly from the CAD group (n = 6) and the NCAD group (n = 6), respectively. Figure 1A showed the representative slides of EAT and SAT from one patient with CAD (Figure 1A-a and 1A-b) and one without CAD (Figure 1A-c and 1A-d). Immunohistochemical staining revealed that chemerin was expressed in both EAT and SAT of the two groups. Furthermore, as shown in Figure 1B, quantitative analysis of immunohistochemistry revealed that the amounts of chemerin protein in EAT were higher in the patients with CAD than those without CAD (70128.28 ± 13068.83 vs. 52312.03 ± 9899.90, *P *< 0.05). For the CAD group, significantly higher level of chemerin protein was found in EAT than in SAT (70128.28 ± 13068.83 vs. 42942.04 ± 15460.67, *P *< 0.01), whereas no significant difference was found between EAT and SAT in the NCAD group (52312.03 ± 9899.90 vs. 50533.71 ± 17289.54, *P *= 0.871).

### EAT adipokines mRNA expression between CAD group and NCAD group

**Figure 2:**
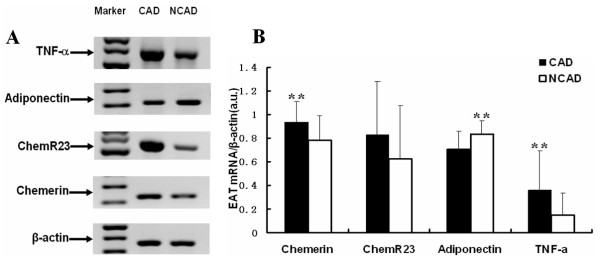
**The mRNA expression levels of adipokines in EAT between the two groups**. (A): the result of agarose gel electrophoresis of PCR products in EAT. (B): the result of relative mRNA levels of chemerin, chemR23, adiponectin and TNF-α in EAT of the two groups (CAD group, n = 37; NCAD group, n = 16). ** indicates *P *< 0.01, CAD group vs. NCAD group. Abbreviations: a.u., arbitrary units.

As illustrated in Figure 2, we found significantly higher levels of chemerin, TNF-α mRNA and significantly lower level of adiponectin mRNA in EAT of CAD group compared to that of NCAD group, whereas the mRNA expression of chemR23 in EAT did not show significant difference between the two groups (chemerin 0.94 ± 0.17 vs. 0.78 ± 0.21, *P *< 0.01; TNF-α 0.36 ± 0.34 vs. 0.15 ± 0.19, *P *< 0.01; adiponectin 0.71 ± 0.15 vs. 0.83 ± 0.12, *P *< 0.01; chemR23 0.83 ± 0.45 vs. 0.62 ± 0.45, *P *= 0.140).

### Chemerin mRNA expression between EAT and SAT

**Figure 3:**
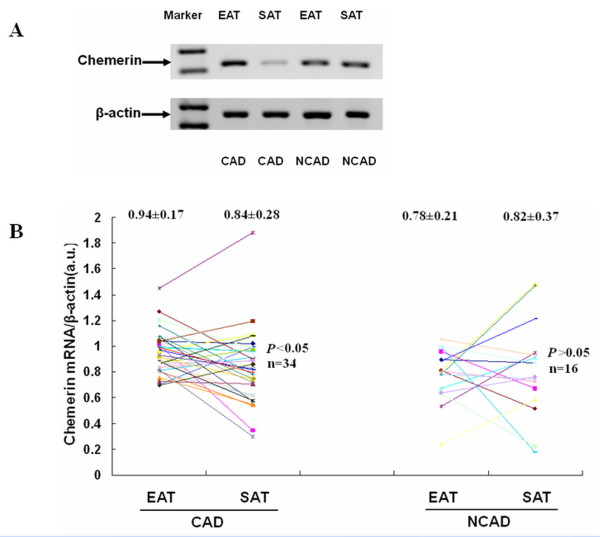
**The mRNA expression level of chemerin between EAT and SAT**. (A): the result of agarose gel electrophoresis of PCR products in EAT and SAT. (B): the scattered graph of relative mRNA levels of chemerin in EAT and SAT of the two groups (CAD group, n = 34; NCAD group, n = 16).

As for the comparison of paired EAT and SAT, 3 SAT samples of CAD group were excluded from the analysis due to insufficient adipose tissue biopsy samples or being damaged during the operation. As shown in Figure 3, in the CAD group, significantly higher level of chemerin mRNA was observed in EAT than in paired SAT (0.94 ± 0.17 vs. 0.84 ± 0.28, *P *< 0.05), whereas chemerin mRNA level did not seem to differ between the two adipose depots in the NCAD group (0.78 ± 0.21 vs. 0.82 ± 0.37, *P *> 0.05). Moreover, chemerin mRNA expression in SAT of CAD group was comparable with that in SAT of NCAD group (0.84 ± 0.28 vs. 0.82 ± 0.37, *P *> 0.05).

### Association of EAT chemerin mRNA expression with clinical parameters

Chemerin mRNA expression in EAT was positively correlated with Gensini score (r = 0.365, *P *< 0.05). Furthermore, this correlation revealed by partial correlation analysis remained statistically significant (r = 0.357, *P *< 0.05) even after adjusting for age, gender, BMI and waist circumference. Chemerin mRNA expression in EAT was also found to be positively correlated with BMI (r = 0.305, *P *< 0.05), waist circumference (r = 0.384, *P *< 0.01), fasting glucose (r = 0.334, *P *< 0.05), chemR23 mRNA expression in EAT (r = 0.349, *P *< 0.05) and negatively correlated with adiponectin mRNA expression in EAT (r = -0.322, *P *< 0.05). However, no association was observed between chemerin mRNA expression in EAT and TNF-α mRNA expression in EAT (*P *> 0.05).

### Serum adipokines analysis

The serum samples of 33 CAD patients and 11 NCAD patients met the criteria for analysis. The two groups were also age and gender matched. There were no significant differences in the serum levels of chemerin or adiponectin between the two groups (chemerin 118.13 ± 32.02 ng/ml vs. 99.60 ± 28.66 ng/ml, *P *= 0.096; adiponectin 4.90 ± 1.39 μg/ml vs. 5.87 ± 1.52 μg/ml, *P *= 0.155). Correlation analysis revealed that serum chemerin concentration was positively correlated with BMI (r = 0.323, *P *< 0.05), waist circumference (r = 0.398, *P *< 0.01), HOMA-IR (r = 0.299, *P *< 0.05) and hsCRP (r = 0.340, *P *< 0.05). However, neither serum chemerin (r = 0.295, *P *= 0.120) nor serum adiponectin (r = -0.328, *P *= 0.083) level was found to be associated with Gensini score. Also, there was no statistically significant correlation between serum chemerin level and its expression level in EAT (*P *> 0.05).

## Discussion

In the present study, we found that EAT of CAD group showed significantly higher levels of chemerin mRNA and protein when compared with that of NCAD patients in Han Chinese patients. Moreover, chemerin mRNA expression in EAT was positively correlated with the severity of coronary atherosclerosis. However, there was no significant difference in the serum level of chemerin between the two groups. Likewise, no statistically significant association was found between serum chemerin level and coronary atherosclerosis. Spiroglou et al. have reported a positive correlation between coronary atherosclerosis and chemerin expression in pericoronary adipose tissue of autopsy cases [17], but their research was not a case-control study, and whether the expression of chemerin was affected by the diseases that subjects suffered or by death were uncertain. Our study excluded the established confounding factors which might influence the expression of chemerin and demonstrated the correlation of chemerin expression in EAT and the coronary atherosclerosis in Han Chinese patients, which was consistent with the results of the study by Spiroglou et al. Another study found serum chemerin level was not associated with coronary atherosclerotic plaque morphology as determined by computed tomography-angiography in patients with stable chest pain [15], which was consistent with our findings. Most recently, Becker and his co-workers reported that long-term overexpression of chemerin did not significantly affect extent of atherosclerotic lesion area in vivo [18]. Taken together, all these studies suggested that locally produced chemerin by EAT rather than its circulating level might affect the atherosclerotic process.

As expected, we observed that both chemerin mRNA expression in EAT and its serum level were positively correlated with indexes of obesity (BMI and waist circumference). The present findings were to some extent supported by the fact that significantly higher chemerin secretion from adipose tissue [19] as well as its circulating level in obese patients [20] and significantly decreased serum chemerin level with weight loss after bariatric surgery [21]. With regard to the close association of chemerin and obesity, a plausible interpretation was that chronic low-grade inflammation that occurred in obesity might promote chemerin production by adipocytes [22,23]. On the other hand, chemerin could also promote the differentiation of preadipocytes in vitro [24], thereby might contribute to obesity.

Previous studies have reported that chemerin mRNA expression in EAT was associated with the indexes of obesity, and obesity has been considered as one of the most important risk factors for CAD. In order to exclude the effects of these potential confounders on coronary atherosclerosis, we performed partial correlation analysis and observed the correlation between chemerin mRNA expression in EAT and coronary atherosclerosis remained statistically significant after adjusting for age, gender, BMI and waist circumference. The association between EAT-derived chemerin and coronary atherosclerosis could be explained from two aspects below. First, significantly increased levels of inflammatory cytokines (TNF-α and IL-1β) in circulating and coronary blood of patients with CAD could up-regulate chemerin in adjacent EAT via "inside to outside" signaling [22,23]. On the other hand, as *in vitro *studies indicated, chemerin could recruit and activate macrophages [25], promote cholesterol uptake [26] and induce endothelial angiogenesis in vitro [27], so we supposed that *in vivo *chemerin secreted by EAT could also be transported downstream to interact with each layer of the coronary wall through "outside to inside" signaling [28], thus participating in several stages of the coronary atherosclerotic process: chemotaxis, foam cell formation and plaque destabilization. Hence we hypothesized that EAT-derived chemerin and coronary atherosclerosis might interact with each other. However, because our research was a cross-sectional study, we could not draw any causal conclusions about their association. The direct effects of EAT-derived chemerin on coronary atherosclerosis *in vivo *still needs to be further ascertained.

Previous studies have highlighted that significantly lower expression of adiponectin [7,29] and significantly higher expression of chemokine (MCP-1) and several inflammatory cytokines (TNF-α, IL-1β, IL-6) were observed in EAT than in SAT [28]. In our study, we also found chemerin mRNA expression was significantly higher in EAT compared to paired SAT for CAD patients, whereas for NCAD patients, no significant difference was found between the two adipose depots. This result indicated that EAT might play a more significant role in the pathogenesis of coronary atherosclerosis than SAT.

We also concluded that EAT of CAD group showed significantly lower expression of adiponectin and significantly higher expression of TNF-α when compared with that of NCAD patients. This finding was confirmed by previous studies [6,7], suggested the pro- and anti-inflammatory unbalance in EAT of patients with CAD. In addition, we observed a negative correlation between chemerin mRNA expression in EAT and adiponectin mRNA expression in EAT, which was consistent with the correlation of their protein levels [17].

Just as Mazurek et al. [28] illustrated that local inflammatory burden in EAT did not correlate with their circulating concentrations, in the present study, no association was found between serum chemerin level and its expression level in EAT. The following reasons could be put forward to explain this phenomenon. First, we merely detected chemerin at the transcription level which might not reflect the level of its protein translation and secretion. Second, EAT is only 1% of total fat mass, apart from EAT, other adipose depots as well as liver were also important sources of circulating chemerin level [24,30]. Therefore, the contribution of EAT-derived chemerin to its systemic level might be negligible.

Several limitations of the present study should be considered. First, owing to the restriction of the amount of EAT biopsy samples, we merely detected chemerin mRNA and protein in EAT. It might have been useful to measure the level of chemerin secretion from EAT. Second, although the sample size of our study was relatively large in comparison with other similar studies on EAT, we still could not carry out multivariate analysis to further clarify whether overexpression of chemerin in EAT was an independent risk factor for coronary atherosclerosis. Finally, for serum analyses, the small serum sample size as well as the absence of serums of normal controls might have prevented us from achieving statistically significant associations.

## Conclusions

In conclusion, our study demonstrates that the expressions of chemerin mRNA and protein are significantly higher in EAT from patients with CAD in Han Chinese patients. Furthermore, the severity of coronary atherosclerosis is associated with the level of chemerin mRNA in EAT rather than its circulating level. Further studies aiming at exploring the direct effects of EAT-derived chemerin on coronary atherosclerosis and its related molecular mechanisms will provide a novel target for the prevention and treatment of CAD.

## Competing interests

The authors declare that they have no competing interests.

## Authors' contributions

XG carried out the molecular genetic studies and serum detection, participated in the design of the study and drafted the manuscript. FZ carried out the immunoassays. XG, FZ and XZ involved in the acquisition of data as well as analysis and interpretation of data. YL and FG collected adipose biopsy samples and serum samples. XG performed the statistical analysis. HT and LW have been involved in revising the manuscript critically for important intellectual content. HT, SM and FG conceived of the study, and participated in its design and coordination. All authors have read and approved the final manuscript.
